# Dietary Supplement Use and Colorectal Adenoma Risk in Individuals with Lynch Syndrome: The GEOLynch Cohort Study

**DOI:** 10.1371/journal.pone.0066819

**Published:** 2013-06-18

**Authors:** Renate C. Heine-Bröring, Renate M. Winkels, Akke Botma, Fränzel J. B. van Duijnhoven, Audrey Y. Jung, Jan H. Kleibeuker, Fokko M. Nagengast, Hans F. A. Vasen, Ellen Kampman

**Affiliations:** 1 Division of Human Nutrition, Wageningen University, Wageningen, The Netherlands; 2 National Institute for Public Health and the Environment (RIVM), Bilthoven, The Netherlands; 3 Department of Epidemiology, Biostatistics, and HTA, Radboud University Nijmegen Medical Centre, Nijmegen, The Netherlands; 4 Department of Gastroenterology, University Medical Center Groningen, University of Groningen, Groningen, The Netherlands; 5 Department of Gastroenterology, Radboud University Nijmegen Medical Centre, Nijmegen, The Netherlands; 6 Netherlands Foundation for the Detection of Hereditary Tumours, Leiden, The Netherlands; 7 Department for Health Sciences, VU University Amsterdam, Amsterdam, The Netherlands; Sookmyung Women's University, Republic Of Korea

## Abstract

**Background and Aims:**

Individuals with Lynch syndrome have a high lifetime risk of developing colorectal tumors. In this prospective cohort study of individuals with Lynch syndrome, we examined associations between use of dietary supplements and occurrence of colorectal adenomas.

**Materials and Methods:**

Using data of 470 individuals with Lynch syndrome in a prospective cohort study, associations between dietary supplement use and colorectal adenoma risk were evaluated by calculating hazard ratios (HR) and 95% confidence intervals (CI) using cox regression models adjusted for age, sex, and number of colonoscopies during person time. Robust sandwich covariance estimation was used to account for dependency within families.

**Results:**

Of the 470 mismatch repair gene mutation carriers, 122 (26.0%) developed a colorectal adenoma during an overall median person time of 39.1 months. 40% of the study population used a dietary supplement. Use of any dietary supplement was not statistically significantly associated with colorectal adenoma risk (HR = 1.18; 95%CI 0.80–1.73). Multivitamin supplement use (HR = 1.15; 95%CI 0.72–1.84), vitamin C supplement use (HR = 1.57; 95%CI 0.93–2.63), calcium supplement use (HR = 0.69; 95%CI 0.25–1.92), and supplements containing fish oil (HR = 1.60; 95%CI 0.79–3.23) were also not associated with occurrence of colorectal adenomas.

**Conclusion:**

This prospective cohort study does not show inverse associations between dietary supplement use and occurrence of colorectal adenomas among individuals with Lynch syndrome. Further research is warranted to determine whether or not dietary supplement use is associated to colorectal adenoma and colorectal cancer risk in MMR gene mutation carriers.

## Introduction

Individuals with Lynch syndrome have pathogenic germline mutations in genes involved in DNA mismatch repair (MMR), i.e. MLH1, MSH2, MSH6, PMS2, [1]–[5] or in the EPCAM gene. [6], [7] Approximately 3% of all colorectal cancers are induced by Lynch syndrome. [8], [9] The adenoma-carcinoma sequence seems to be accelerated in MMR gene mutation carriers, [10] and carriers have a 25–70% risk of developing colorectal cancer up to age 70, at a relatively young age, [2]–[5], [11] compared to 2–5% in the general Western population. [12], [13]

Removal of colorectal adenomas lowers risk of colorectal carcinomas in individuals with Lynch syndrome. [14], [15] Therefore, those persons are generally advised to follow strict periodic endoscopic surveillance to detect colorectal adenomas. [15], [16] Considering the high lifetime risk of developing adenomas and carcinomas in MMR gene mutation carriers, [2]–[5] it is very relevant to study whether modifiable lifestyle factors, including dietary supplement use, can affect this risk. As shown in previous studies from our group, excess body weight, [17] smoking, [18] and a dietary pattern high in snack foods [19] were associated with an increased risk of colorectal adenomas in persons with Lynch syndrome. Retrospective case-control studies in Lynch syndrome suspected-families showed that increased fruit consumption and dietary fibre intake possibly decreased the risk of colorectal tumors. [20]

Although a healthy diet provides a sufficient amount of vitamins and minerals, many individuals take vitamin and mineral supplements regularly, hoping to further improve their health and to prevent acute or chronic illnesses and serious diseases, such as cancer. [21], [22] Dietary supplement use and colorectal adenoma risk have been extensively investigated in the general population. No convincing evidence for an association between multivitamin supplement use, [23] folic acid supplement use, [24] and antioxidant supplement use and adenoma occurrence was found, [25], [26] whereas calcium supplement use might contribute to a lower risk of colorectal adenomas. [27] MMR gene mutation carriers might have a higher use of dietary supplements compared to the general Dutch population based on their health status and risk. [28] Up until now, as far as we know, no studies on dietary supplement use and colorectal adenomas among individuals with Lynch syndrome were conducted.

The objective of this study was to prospectively examine the association between the most frequently used dietary supplements and colorectal adenoma development in a cohort study of individuals with Lynch syndrome.

## Materials and Methods

### Ethics Statement

The Medical Ethics Committee of the Radboud University Nijmegen Medical Centre approved the study. All participants gave written informed consent.

### Study population

Individuals with Lynch syndrome participated in the GEOLynch prospective cohort study which was described earlier. [17] Briefly, carriers of a germline mutation in at least one of the mismatch repair genes were identified via linkage to a hereditary tumor registration of the Netherlands Foundation for the Detection of Hereditary Tumors in Leiden, the Radboud University Nijmegen Medical Center in Nijmegen, and the University Medical Center Groningen in Groningen, the Netherlands. Participants had to be between 18 and 80 years of age, Dutch-speaking, white, and mentally competent to participate in this study to be eligible for the study. Terminally ill patients, and those with familial adenomatous polyposis, inflammatory bowel diseases, a complete proctocolectomy or colostomy were excluded.

With approval of their medical specialist, a total of 713 mutation carriers were invited to participate in the study between July 2006 and July 2008. Six hundred ninety-five out of 713 people could be contacted of whom nine were ineligible. Of these, 73% (499 of 686) agreed to participate. We were unable to retrieve medical and personal information from 29 participants. Therefore, a total of 470 participants from at least 161 families were included in this study.

### Exposure assessment

At recruitment, dietary supplement use was collected using a self-administered questionnaire. Information on dietary supplement use included frequency of intake (no intake in the previous month, once a month, 2–3 days a month, once a week, 2–3 days a week, 4–5 days a week, 6–7 days a week), amount of intake (1, 2, 3, 4, or ≥5 tablets, capsules or droplets), and brand name of multivitamins, vitamin C, B-vitamins, folic acid, vitamin D (including vitamin A), vitamin E, calcium, iron, and fish oil supplements. In addition, participants could indicate whether they used other supplements that were not covered by the questionnaire. In this study, users of dietary supplements were defined as those taking any dietary supplement during the last month. When patients took no dietary supplements at all during the last month, they were considered nonusers. Habitual dietary intake was collected using a 183-item self-administered and validated food frequency questionnaire. [29], [30] General lifestyle information was collected with a lifestyle questionnaire containing questions about age, sex, weight, height, smoking habits, medication use, physical activity,[31] family history of colorectal cancer, and medical history.

### Outcome data

Medical information, including information about medical history and colorectal adenomas and carcinomas, was obtained by reviewing medical records regularly from all subjects via the participating centers until information was complete. From every participant, information about previously performed colonoscopies, colorectal surgery, cancer, and adenomatous polyps was gathered before recruitment and during follow up until December 31, 2010. We ascertained detailed information from pathology reports about location, size, and histology for all documented colorectal adenomas that occurred during follow up.

### Data analysis

The outcome of our analysis was the time to diagnosis of the first pathology-confirmed colorectal adenoma. Descriptive statistics were used to describe the demographic characteristics, and characteristics on lifestyle, medical status, dietary supplement use and dietary intake of all 470 MMR gene mutation carriers and from those who were diagnosed with a colorectal adenoma during follow up. In addition, general characteristics were computed for dietary supplement users versus nonusers. Differences in baseline characteristics between users and non-users were tested by the Mann-Whitney U test (continuous variables) or the χ^2^ test (categorical variables).

Cox proportional hazards regression was used to investigate associations of dietary supplement use and colorectal adenoma occurrence. Hazard ratios (HR) with 95% confidence intervals (95% CI) were reported, and robust sandwich covariance estimation was used to account for dependency of observations within families. The Cox proportional hazard models were tested for and met the assumption of proportionality by visually inspecting whether the distance between the log(-log) survival curves was approximately constant. Person time started at the date of the most recent colonoscopy before assessment of dietary supplement use and ended at the date of colonoscopy of the first diagnosed colorectal adenoma during follow up. Participants without a colorectal adenoma diagnosis or without a detectable colorectal adenoma were censored at the date of their last known colonoscopy during follow up.

The following covariates were evaluated as potentially confounding variables: age (continuous), sex, educational level (categorical: high vs lower educated), number of colonoscopies during person time (continuous), history of colorectal adenomas (yes/no), history of carcinomas (yes/no), physical activity level (categorical: high vs lower physically active), smoking status (current, former, never), body mass index (continuous), regular use of NSAIDs (<1 times/week, ≥1 times/week), alcohol intake (g/d), total energy intake (kJ/d), total vegetables intake (g/d), total fruit intake (g/d), and total red meat intake (g/d). In the basic model, we adjusted for age and sex. Covariates were considered as confounders if they correlated with any use of dietary supplements and colorectal tumor risk, and if they changed the hazard ratio by ≥10% using forward selection. The maximally adjusted model included age, sex, and number of colonoscopies during person time.

Stratified analyses for the association of dietary supplement use and colorectal adenoma risk were conducted for MMR carriers with a history of colorectal neoplasms before study entry (recurrence), and for those without (first occurrence). To assess possible effect measure modification of associations between dietary supplement use and colorectal adenoma risk, we stratified our analysis for smoking status (never, former, current) and total fruit and vegetables intake (in quartiles: <179, 179–283, 283–387, ≥387 g/day). Nonusers, who never smoked, were defined as reference group to estimate HRs stratified for smoking status. In addition, nonusers who had a relatively low intake of fruit and vegetables of <179 g/day, were defined as reference group to evaluate HRs stratified for total fruit and vegetables intake. To test for multiplicative interaction we used a log likelihood ratio test, comparing models for nonusers and users by smoking status and by strata of total fruit and vegetables intake.

A sensitivity analysis was performed for the association between any use and use of specific types of dietary supplements and colorectal adenoma risk, in which person time started at the time of assessment of dietary supplement use. P<0.05 was considered statistically significant. All analyses were performed using SAS version 9.2 (SAS Institute Inc., Cary, NC).

## Results

In this cohort of MMR gene mutation carriers, 122 (26.0%) of 470 subjects developed a colorectal adenoma during an overall median person time of 39.1 months (table 1). The total person time was 18,449 person months. About 44% of the colorectal adenoma cases used a dietary supplement versus 40% in the total cohort. Colorectal adenoma cases were slightly older and lower educated compared to the total cohort. In addition, more persons smoked and alcohol intake was higher in colorectal adenoma cases compared to the total cohort.

**Table 1:** General characteristics of the mismatch repair gene mutation carriers in the GEOLynch prospective cohort study.

			Total population	Colorectal adenoma cases
			(n = 470)	(n = 122)
	Person time [months, median (P25–P75)]		39.1 (25.1–49.8)	27.8 (22.4–47.3)
**Demographic**	Age at study entry [years, median (P25–P75)]		50.2 (40.8–58.6)	53.0 (45.7–60.2)
**characteristics**	Women [n (%)]		281 (59.8)	68 (56.0)
	High education [n (%)] ^a^		161 (34.6)	29 (24.0)
**Lifestyle**	BMI [kg/m^2^, median (P25–P75)]		24.5 (22.5–26.9)	25.1 (23.4–27.1)
**characteristics**	Current smokers [n (%)]		85 (18.1)	36 (29.5)
	NSAID use [≥1x/week, n (%)]		42 (9.1)	10 (8.2)
	Physical activity [high; n (%)] ^b^		154 (33.4)	41 (34.5)
**Medical**	MMR gene mutation [n (%)]	MLH1	178 (37.9)	49 (40.2)
**characteristics**		MSH2	192 (40.9)	55 (45.1)
		MSH6	95 (20.2)	17 (13.9)
		PMS2	3 (0.6)	1 (0.8)
	History of cancer [n (%)]	CRC ^c^	121 (25.7)	31 (25.4)
		Other cancer	82 (17.5)	26 (21.3)
	History of colorectal neoplasms [n (%)] ^d^		232 (49.4)	73 (59.8)
	No. of colonoscopies during person time [n (%)]	1	179 (38.1)	70 (57.4)
		2	193 (41.1)	39 (32.0)
		≥3	95 (20.2)	10 (8.2)
**Dietary**	Any [n (%)]		188 (40.0)	54 (44.3)
**supplement use ^e^**	Multivitamins [n (%)]		121 (25.7)	37 (30.3)
	Vitamin C [n (%)]		61 (13.0)	17 (13.9)
	Vitamin B complex [n (%)]		8 (1.7)	1 (0.8)
	Vitamin E [n (%)]		9 (1.9)	1 (0.8)
	Vitamin D [n (%)]		6 (1.3)	1 (0.8)
	Folic acid [n (%)]		9 (1.9)	1 (0.8)
	Calcium [n (%)]		22 (4.7)	6 (4.9)
	Iron [n (%)]		3 (0.6)	0
	Fish oil [n (%)]		32 (6.8)	13 (10.7)
	Other [n (%)] ^f^		58 (12.3)	20 (16.4)
**Dietary intake**	Total energy [kJ/day, mean±SD]		9055±2798	8711±2702
	Vegetables intake [g/day, median (P25–P75)]		123 (76–176)	124 (61–173)
	Fruit intake [g/day, median (P25–P75)]		157 (75–234)	136 (44–234)
	Red meat intake [(g/day, median (P25–P75)]		46 (30–64)	50 (30–67)
	Alcohol intake [g/day, median (P25–P75)]		7.2 (1.5–16.8)	9.4 (3.2–21.8)

a College or university degree

b Highest tertile of the physical activity score

c CRC =  colorectal cancer

d History of colorectal adenoma and/or carcinoma

e Use of any dietary supplement during the last month

f E.g. glucosamine/chondroitin supplements, and garlic pills

Of the dietary supplement users, 28.7% developed a colorectal adenoma during the follow up period (table 2); this was 24.1% in nonusers. Compared to nonusers, dietary supplement users were slightly older, were more often women, were more often higher educated, smoked less often, had more often a history of cancer or colorectal tumors, and consumed more fruit.

**Table 2:** General characteristics of the mismatch repair gene mutation carriers stratified by dietary supplement use in the GEOLynch prospective cohort study.

			Dietary supplement users		
			Users ^a^	Nonusers ^b^	p-value ^h^
			(n = 188)	(n = 282)	
	Person time [months, median (P25–P75)]		36.6 (24.4–49.4)	41.8 (26.0–50.2)	0.17
**Demographic**	Age at study entry [years, median (P25–P75)]		51.7 (42.1–58.6)	48.9 (39.6–58.6)	0.17
**characteristics**	Women [n (%)]		128 (68.1)	153 (54.3)	<0.01
	High education [n (%)] ^c^		70 (37.4)	91 (32.7)	0.59
**Lifestyle**	BMI [kg/m^2^, median (P25–P75)]		24.1 (21.9–26.3)	24.7 (22.9–27.5)	0.23
**characteristics**	Current smokers [n (%)]		31 (16.5)	54 (19.2)	0.15
	NSAID use [≥1x/week, n (%)]		17 (9.2)	25 (9.1)	0.97
	Physical activity [high; n (%)] ^d^		63 (34.2)	91 (32.9)	0.76
**Medical**	MMR gene mutation [n (%)]	MLH1	76 (40.4)	102 (36.2)	0.27
**characteristics**		MSH2	66 (35.1)	126 (44.7)	
		MSH6	43 (22.9)	52 (18.4)	
		PMS2	2 (1.1)	1 (0.4)	
	Colorectal adenoma cases during follow up [n (%)]		54 (28.7)	68 (24.1)	0.28
	History of cancer [n (%)]	CRC ^e^	50 (26.6)	71 (25.2)	0.73
		Other cancer	41 (21.8)	41 (14.5)	0.04
	History of colorectal neoplasms [n (%)] ^f^		99 (52.7)	133 (47.2)	0.31
	No. of colonoscopies during person time [n (%)]	1	69 (36.7)	110 (39.0)	0.83
		2	80 (42.6)	113 (40.1)	
		≥3	37 (19.7)	58 (20.6)	
**Dietary**	Multivitamins [n (%)]		121 (64.4)	-	
**supplement**	Vitamin C [n (%)]		61 (32.5)	-	
**use ^a^**	Vitamin B complex [n (%)]		8 (4.3)	-	
	Vitamin E [n (%)]		9 (4.8)	-	
	Vitamin D [n (%)]		6 (3.2)	-	
	Folic acid [n (%)]		9 (4.8)	-	
	Calcium [n (%)]		22 (11.7)	-	
	Iron [n (%)]		3 (1.6)	-	
	Fish oil [n (%)]		32 (17.0)	-	
	Other [n (%)] ^g^		58 (30.9)	-	
**Dietary intake**	Total energy intake [kJ/day, mean±SD]		8871±2647	9179±2893	0.36
	Vegetables intake [g/day, median (P25–P75)]		123 (81–177)	125 (73–175)	0.61
	Fruit intake [g/day, median (P25–P75)]		167 (79–235)	130 (53–233)	0.11
	Red meat intake [(g/day, median (P25–P75)]		46 (27–63)	46 (34–64)	0.38
	Alcohol intake [g/day, median (P25–P75)]		6.7 (1.6–16.3)	7.4 (1.5–17.1)	0.69

a Use of any dietary supplement during the last month

b No use of dietary supplement during the last month

c College or university degree

d Highest tertile of the physical activity score [31]

e CRC =  colorectal cancer

f History of colorectal adenoma and/or carcinoma

g E.g. glucosamine/chondroitin supplements, and garlic pills

h Calculated using Mann-Whitney U test for continuous variables or the χ2 test for categorical variables

Use of any dietary supplement was not statistically significantly associated with colorectal adenoma risk (HR = 1.18; 95% CI 0.80–1.73) after adjustments for age, sex, and number of colonoscopies during person time (table 3). In addition, no associations were found between colorectal adenomas and multivitamin supplement use (HR = 1.15; 95% CI 0.72–1.84), vitamin C supplement use (HR = 1.57; 95% CI 0.93–2.63), calcium supplement use (HR = 0.69; 95% CI 0.25–1.92), and supplements containing fish oil (HR = 1.60; 95% CI 0.79–3.23).

**Table 3:** Association of dietary supplement use and colorectal adenoma risk in the GEOLynch prospective cohort study of MMR gene mutation carriers.

Dietary supplement use		No use ^a^	Use ^b^
		HR	HR (95% CI)
Any dietary supplement	No. of cases/non-cases	68/214	54/134
	Person time (months, median)	41.8	36.6
	HR, adjusted for age & sex	1.0	1.21 (0.85–1.72)
	HR, adjusted for age, sex, and number of colonoscopies during person time	1.0	1.18 (0.80–1.73)
			
Multivitamins	No. of cases/non-cases	85/264	37/84
	Person time (months, median)	39.6	37.5
	HR, adjusted for age & sex	1.0	1.38 (0.93–2.07)
	HR, adjusted for age, sex, and number of colonoscopies during person time	1.0	1.15 (0.72–1.84)
			
Vitamin C	No. of cases/non-cases	105/304	17/44
	Person time (months, median)	39.9	34.5
	HR, adjusted for age & sex	1.0	1.36 (0.80–2.31)
	HR, adjusted for age, sex, and number of colonoscopies during person time	1.0	1.57 (0.93–2.63)
			
Calcium	No. of cases/non-cases	116/332	6/16
	Person time (months, median)	39.2	36.9
	HR, adjusted for age & sex	1.0	0.68 (0.24–1.93)
	HR, adjusted for age, sex, and number of colonoscopies during person time	1.0	0.69 (0.25–1.92)
			
Fish oil	No. of cases/non-cases	109/329	13/19
	Person time (months, median)	39.2	37.1
	HR, adjusted for age & sex	1.0	1.74 (1.00–3.01)
	HR, adjusted for age, sex, and number of colonoscopies during person time	1.0	1.60 (0.79–3.23)

a No use of dietary supplements during the last month

b Use of any dietary supplement during the last month

In this study population, 73 of the 232 MMR carriers with a history of colorectal neoplasms before study entry developed a colorectal adenoma during follow up, of which 36 were dietary supplement user. In MMR carriers without a history of colorectal neoplasms (n = 238), 49 subjects developed a colorectal adenoma of which 18 subjects used a dietary supplement. Among MMR carriers with a history of colorectal neoplasms, dietary supplement use was not statistically significantly associated with colorectal adenoma risk (HR = 0.87; 95% CI 0.44–1.75). However, a borderline statistically significantly increased risk was observed for dietary supplement use and colorectal adenoma risk among those without a history of colorectal neoplasms (HR = 1.60; 95% CI 0.98–2.60).

Table 4 shows that there is no effect measure modification with smoking status (p for multiplicative interaction: 0.41) and total fruit and vegetables intake (p for multiplicative interaction: 0.39) in the association of dietary supplement use and colorectal tumor risk. In a sensitivity analysis, in which person time started at the time of assessment of dietary supplement use, no differences in associations were observed for any use or use of specific types of dietary supplements and colorectal adenoma risk. However, a borderline statically significantly increased risk of colorectal adenomas was observed for persons who took supplements containing fish oil (HR = 1.78; 95% CI 0.92–3.45).

**Table 4:** Association of any dietary supplement use and colorectal adenoma risk stratified for smoking status and total fruit and vegetables intake in the GEOLynch prospective cohort study of MMR gene mutation carriers.

	Smoking status			
Dietary supplement use	Never	Former	Current	
*No use^a^*				
No. of cases/non-cases	17/99	28/83	23/31	
Person time (months, median)	46.0	44.9	31.0	
HR (95% CI) adjusted for age, sex, and number of colonoscopies during person time	1.0	1.54 (0.84–2.84)	2.54 (1.42–4.54)	
*Use^b^*				
No. of cases/non-cases	8/56	33/60	13/18	
Person time (months, median)	37.2	35.9	39.1	
HR (95% CI) adjusted for age, sex, and number of colonoscopies during person time	0.89 (0.39–2.00)	2.05 (1.10–3.82)	3.37 (1.59–7.13)	
P for multiplicative interaction				0.41

	Total fruit and vegetables intake (g/day)			
Dietary supplement use	<179	179–283	283–387	≥387
*No use^a^*				
No. of cases/non-cases	26/51	10/60	15/51	18/51
Person time (months, median)	34.3	45.9	45.3	36.8
HR (95% CI) adjusted for age, sex, and number of colonoscopies during person time	1.0	0.48 (0.23–1.03)	0.88 (0.44–1.74)	0.78 (0.45–1.35)
*Use^b^*				
No. of cases/non-cases	16/25	13/34	14/38	14/34
Person time (months, median)	27.2	42.1	40.3	35.9
HR (95% CI) adjusted for age, sex, and number of colonoscopies during person time	1.25 (0.63–2.52)	0.97 (0.45–2.08)	0.96 (0.45–2.03)	0.66 (0.29–1.50)
P for multiplicative interaction				0.39

a No use of dietary supplements during the last month

b Use of any dietary supplement during the last month

## Discussion

The present study did not observe a statistically significant association between use of any dietary supplement and colorectal adenoma risk among individuals with Lynch syndrome. No marked associations were found for multivitamin supplement use, vitamin C supplement use, calcium supplement use, and use of supplements containing fish oil and colorectal adenoma risk either.

To our best knowledge, this is the first prospective cohort study that examined the association of dietary supplement use and development of colorectal adenomas in individuals with Lynch syndrome. The association between dietary supplement use and sporadic colorectal adenoma risk in the general population has been investigated in many epidemiological studies. [23]–[27] Our findings for dietary supplement use and colorectal adenoma risk in individuals with Lynch syndrome were largely consistent with findings in the general population. Calcium supplement use might contribute to a lower risk of colorectal adenomas in the general population. [27] However, no evidence of association was found for calcium supplement use and colorectal adenoma risk in this Lynch syndrome population. A randomized, double-blind, placebo-controlled trial with calcium supplements, conducted in 30 first-degree relatives of Lynch syndrome patients, showed a small but nonstatistically significant reduction in epithelial cell proliferation in biopsies of the rectum, and no effect in the sigmoid and descending colon compared with placebo after 12 weeks of intervention. [32] Those results also do not suggest that the use of calcium supplements may help to lower the increased risk of colorectal adenoma occurrence among mismatch repair gene carriers.

No significant association was shown for fish oil supplements and colorectal adenoma risk, when person time started at the date of the most recent colonoscopy before assessment of dietary supplement use. However, according to sensitivity analyses when person time started at the time of assessment of dietary supplement use, a borderline statistically significantly increased risk for colorectal adenomas was observed for Lynch syndrome patients who took fish oil supplements. Our findings should be interpreted with caution, as our hazard ratios appear to be unstable, probably due to the low number of fish oil supplement users in our study. Moreover, the possible detrimental role of supplements containing fish oil on colorectal tumor risk in Lynch syndrome patients contrasts with findings in the general population: fish oil, and then particularly n-3 PUFA from fish oil, are thought to play a beneficial role in the prevention of colorectal cancer due to its anti-angiogenic and anti-inflammatory properties and its regulatory role in cell proliferation and apoptosis. [33]–[36] Nevertheless, the potentially increased risk for developing colorectal adenomas due to supplements containing fish oil in our study corresponds with findings in a nested case-control study within the VITamins And Lifestyle cohort. High intake of n-3 PUFA from diet plus supplements was associated with a decreased risk of colorectal cancer among those at low genetic risk (HR  =  0.23; 95% CI: 0.07–0.78), while such intake was associated with a substantially increased risk among those at high genetic risk (HR =  5.79; 95% CI: 1.79–18.7). Genetic risk was calculated using a genetic risk score enumerating the number of risk alleles present at 16 single nucleotide polymorphisms (SNPs) located within known/recently-identified CRC susceptibility loci (Personal communication, Elizabeth Kantor). Expansion of our Lynch syndrome cohort and a longer follow up are essential to further investigate the role of fish oil supplements and colorectal adenoma risk.

Stratified analyses for MMR carriers with and without a history of colorectal neoplasms before study entry showed no association for dietary supplement use and colorectal adenoma risk among those without a history of colorectal neoplasms, whereas a borderline statistically significantly increased risk was observed for MMR carriers with a history of colorectal neoplasms. Due to the low number of users reflected in the wide confidence intervals in this study, we are not able to draw firm conclusions considering differences between these two groups.

Several critical points regarding the study design and dietary supplement use need to be highlighted. The combined analysis of all the different kinds of dietary supplements on colorectal adenoma risk may mask individual effects of each nutrient supplement. Although this study is the largest prospective study in MMR gene mutation carriers up until now, the small number of users of individual dietary supplements in our study narrows the extent to which we can observe associations.

MMR gene mutation carriers might have increased their dietary supplement intake based on their health status and risk, [28] and may therefore have a higher use of dietary supplements compared to the general Dutch population. However, dietary supplement use in this Lynch syndrome population was similar (40%) to the general Dutch population (30–56%); also in our study dietary supplement users were more often women. [37] Moreover, we relied on self-reporting which makes misclassification of dietary supplement use possible. However, according to several studies, self-reported dietary supplement use is a reliable method to measure intake of dietary supplements. [38]–[40] Thus, dietary supplement use in individuals with Lynch syndrome included in this study reflects use in the general Dutch population and is a reliable indicator for the actual intake.

As information on dosage and duration was not assessed, we could not calculate the total nutrient intake by foods and dietary supplements together, and were unable to examine changes in dietary supplement use over time. Our data only allowed us to examine the associations for dietary supplement use as it was reported before the events of interest.

Strengths of this study are the inclusion of confirmed MMR gene mutation carriers in our cohort, and the high participation rate of 73%. These factors make our findings generalizable to Lynch syndrome patients in comparable clinical settings. Other strengths are the prospective cohort design, the relatively long person time, and the ability to adjust for many potential confounders.

In conclusion, in this prospective cohort study no associations between dietary supplement use and colorectal adenoma risk among individuals with Lynch syndrome were indicated. Further research is warranted to determine whether or not dietary supplement use is associated to colorectal adenoma and colorectal cancer risk in MMR gene mutation carriers.
