# Correction: Dietary Supplement Use and Colorectal Adenoma Risk in Individuals with Lynch Syndrome: The GEOLynch Cohort Study

**DOI:** 10.1371/annotation/be050d56-4aeb-403b-835f-135c7e5f83b2

**Published:** 2014-01-02

**Authors:** Renate C. Heine-Bröring, Renate M. Winkels, Akke Botma, Fränzel J. B. van Duijnhoven, Audrey Y. Jung, Jan H. Kleibeuker, Fokko M. Nagengast, Hans F. A. Vasen, Ellen Kampman

There was an error in the third sentence, of the third paragraph, under the subheading 'Data analysis' of the Materials and Methods. The correct sentence is: 

Covariates were considered as confounders if they correlated with any use of dietary supplements and colorectal adenoma risk, and if they changed the hazard ratio by ≥10% using forward selection.

There is an error in reference 18. The correct reference is: 

Winkels RM, Botma A, Van Duijnhoven FJ, Nagengast FM, Kleibeuker JH, et al. (2012) Smoking Increases the Risk for Colorectal Adenomas in Patients With Lynch Syndrome. Gastroenterology. 142: 241-247

There is an error in reference 28. The correct reference is: 

Carroll C, Booth A, Cooper K (2011) A worked example of "best fit" framework synthesis: A systematic review of views concerning the taking of some potential chemopreventive agents. Bmc Medical Research Methodology 11: 29

